# The advanced concepts for septal l-strut re-designing in septorhinoplasty for better strength and stability by considering of center of gravity

**DOI:** 10.1371/journal.pone.0288607

**Published:** 2023-07-17

**Authors:** Patcharaporn Wongchadakul, Suphalerk Lohasammakul, Phadungsak Rattanadecho, Sorawuth Chu-Ongsakul

**Affiliations:** 1 Princess Srisavangavadhana College of Medicine, Chulabhorn Royal Academy, Bangkok, Thailand; 2 Department of Anatomy, Faculty of Medicine, Siriraj Hospital, Mahidol University, Bangkok, Thailand; 3 Center of Excellence in Electromagnetic Energy Utilization in Engineering (C.E.E.E.) Department of Mechanical Engineering, Faculty of Engineering, Thammasat University (Rangsit Campus), Pathumthani, Thailand; 4 Division of Plastic and Reconstructive Surgery, Faculty of medicine, Siriraj Hospital, Bangkok, Thailand; University of Catania, ITALY

## Abstract

**Purpose:**

This study contributes to the multidisciplinary understanding of septal L-strut reshaping and introduces innovative surgical design concepts based on engineering principles of static equilibrium. The objective is to enhance structural strength and stability, ultimately leading to improved surgical outcomes.

**Method:**

Finite element analysis is employed to model the three-dimensional septal cartilage in septoplasty. A significant contribution of this work is the introduction of an innovative redesigns for the septal L-strut structure. These redesigns represent the first-ever attempt to incorporate the center of gravity theory into the modeling of the septal L-strut.

**Results:**

Our findings emphasize the significance of attaining a lower center of gravity in the design of the septal L-strut, as it contributes to optimal core strength and stability. To achieve this, we recommend widening the caudal septum and shaping the interior fillet corner to its maximum size, taking into account its specific shape. Notably, the utilization of a standard 20x20 mm septal L-strut, the C-shaped technique, and the septal support graft technique provide superior strength due to enhanced basement support.

**Conclusion:**

To enhance surgical outcomes in septal L-strut procedures, design modifications are proposed to improve strength and stability, resulting in optimized performance. Recommendations include widening the caudal septum and incorporating fillet shapes in the geometry to lower the center of gravity.

## Introduction

**Fig 1:**
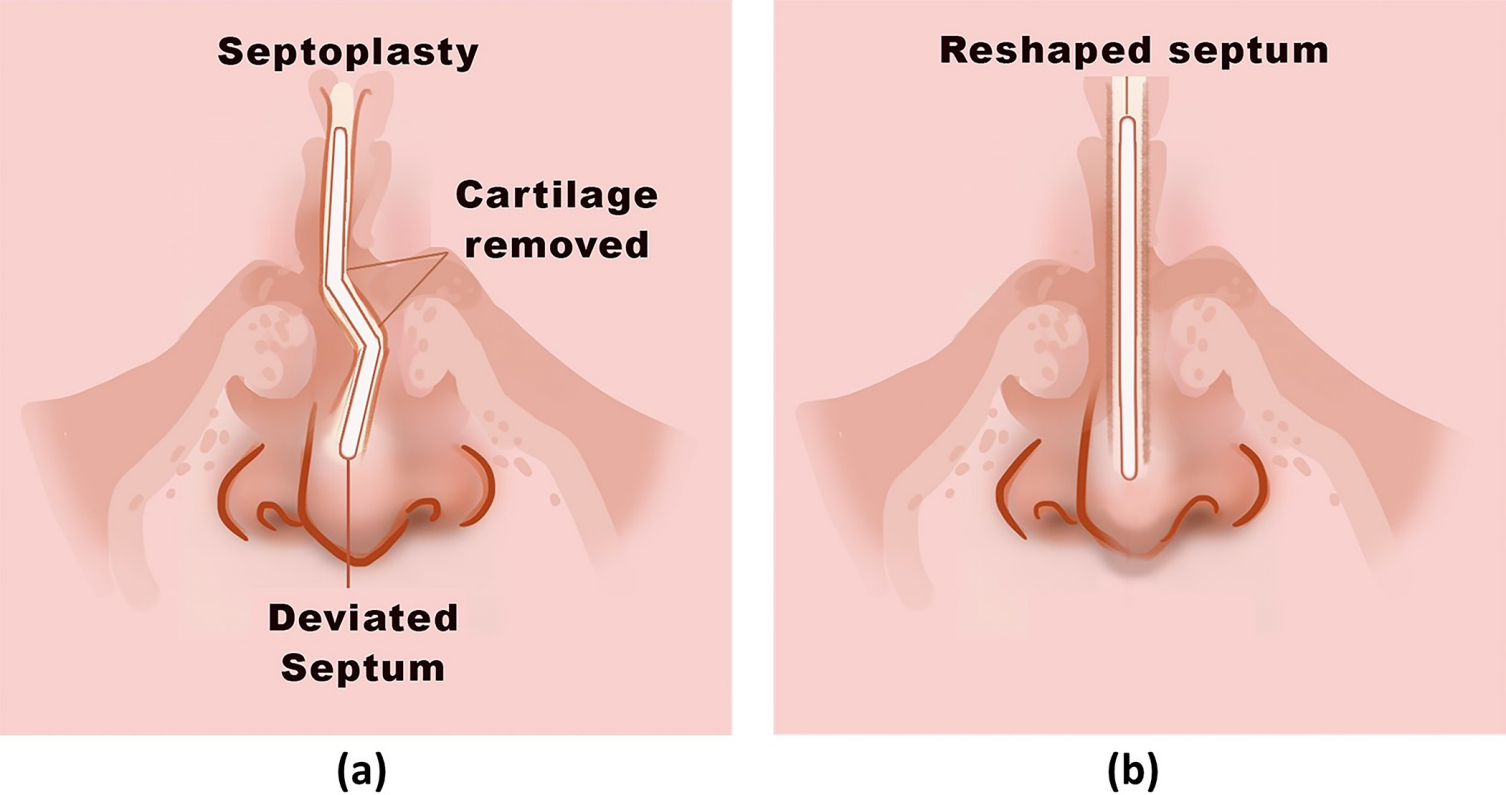
(a) Deviated nasal septum (b) Reshaped nasal septum.

The nasal septum, positioned in the middle of the nose, plays a critical role in maintaining the structural integrity and stability of the nose. Structurally, the cartilaginous nasal septum acts as a supporting beam and cantilever, providing essential support for the nose [1]. Complications such as saddle nose and nasal tip ptosis can occur when this support is weakened [2]. Deviated nasal septum (Fig 1(A)) is a common condition where the nasal partition is misaligned, resulting in nasal obstruction and impacting both nasal function and appearance [3–6]. Its causes can be congenital and/or traumatic [7].

Septoplasty is a surgical procedure aimed at correcting a deviated nasal septum. It offers multiple beneficial improvements, including alleviating nasal obstruction and related conditions, providing nasal support, and enhancing aesthetic outcomes [8, 9]. The main principle of this procedure involves reshaping and/or removing the central portion of the deviated cartilaginous septum, resulting in the formation of the septal L-strut, which consists of the preserved dorsal and caudal septum. The septal L-strut provides support and stability to the nose [10–12].

Excessive removal of cartilage during septoplasty can lead to the aforementioned complications, necessitating revision procedures [13]. These complications primarily arise from a loss of stability in the remaining septal L-strut, which is essential for adequately supporting the nasal structures [14]. Understanding the anatomical and physiological changes in the cartilaginous septum, particularly its ability to withstand loads within the residual septal L-strut, is crucial. This understanding plays a significant role in preventing complications and minimizing the need for additional procedures.

This study combines the expertise of multiple disciplines, including engineering and medicine, to address the challenges in septoplasty. To achieve this, computational modeling techniques using finite element analysis have been employed. This approach allows us to predict and analyze the behavior of the septal L-strut under reaction forces, taking into account its structural characteristics and principles of static equilibrium, such as the center of gravity. Predicting the physical behavior of the septal L-strut in this study provides guidance for surgical procedures and enhances understanding of the internal forces within its structure. Additionally, it offers a visual estimation of the physical stability of the septal L-strut [15], contributing to the improvement of nasal structure design.

Optimizing surgical outcomes depends on strategically designing the septal L-strut, while employing the principle of center of gravity, which focuses on lowering the center of gravity to enhance strength and stability. This study introduces a groundbreaking approach by applying the center of gravity theory to model the septal L-strut, representing the first attempt to introduce an innovative redesign of its structure. The objectives of this research are as follows: (1) Investigating the impact of structural factors, such as widths, cutting angles, and corner shapes. (2) Proposing a novel surgical design based on engineering principles of static equilibrium, aiming to preserve the integrity and stability of the residual septal L-strut. (3) Reducing the revision rate of surgery. (4) Providing a multidisciplinary understanding of the L-strut structure and its strength, offering insights that can be applied to other design concepts for achieving better outcomes.

## Formulation of the problem

The septal L-strut is formed through partial removal of the septal cartilage [16]. To enhance surgical outcomes, it is crucial to optimize the structure of the septal L-strut, thereby improving its strength and stability. This study focuses on investigating the effects of various septal L-strut geometries, including the dimensions of the caudal and dorsal portions, the interior corner shape, and the cutting angle. Previous techniques, such as the C-shaped septal strut, have been proposed to enhance its structural strength [17]. In this work, these techniques are computationally studied and compared to the standard approach, evaluating their structural performance. Furthermore, this study introduces a novel surgical technique called the "septal support graft," which is derived from the septal extension graft technique [18, 19].

The rule of engineering static equilibrium is taken into account in order to develop septal L-strut design, accordingly. The principle of center of gravity is important for a structure to maintain its stability [15]. The object will eventually topple over when center of gravity locates outside its base. Lower position of gravitational center results in a better structural stability due to a smaller degree of relocation after temporary deformation than one with higher position. Thus, increasing the basement area will shift the center of gravity to a more favorable position and improve object’s stability [20]. Step-by-step experiments from obtaining mass and center of gravity to re-designing the object is crucial in complicated-shape object [21, 22].

## Methods and model

The study focuses on design improvement by studying mechanical deformation behavior of varieties of septal L-strut geometry. The numerical model of septal cartilage in rhinoplasty surgery under loading force is performed by COMSOL Multiphysics software with finite element method, with the same condition of tip displacement under loading force of 20 g [23]. The models of septal L-strut are created by SOLIDWORK. The sets of stress, strain and displacement equations are calculated with the mechanical properties of septal cartilage bone, density, Young’s Modulus and Poisson’s ratio as be seen in Table 1 [24–27].

**Table 1:** Mechanical properties of septal cartilage bone.

Mechanical properties of septal cartilage bone	
Young’s Modulus	1 MPa [20]
Poisson’s ration	0.45 [21]
Density	1100 kg/m^3^ [18, 19]

### Physical model

**Fig 2:**
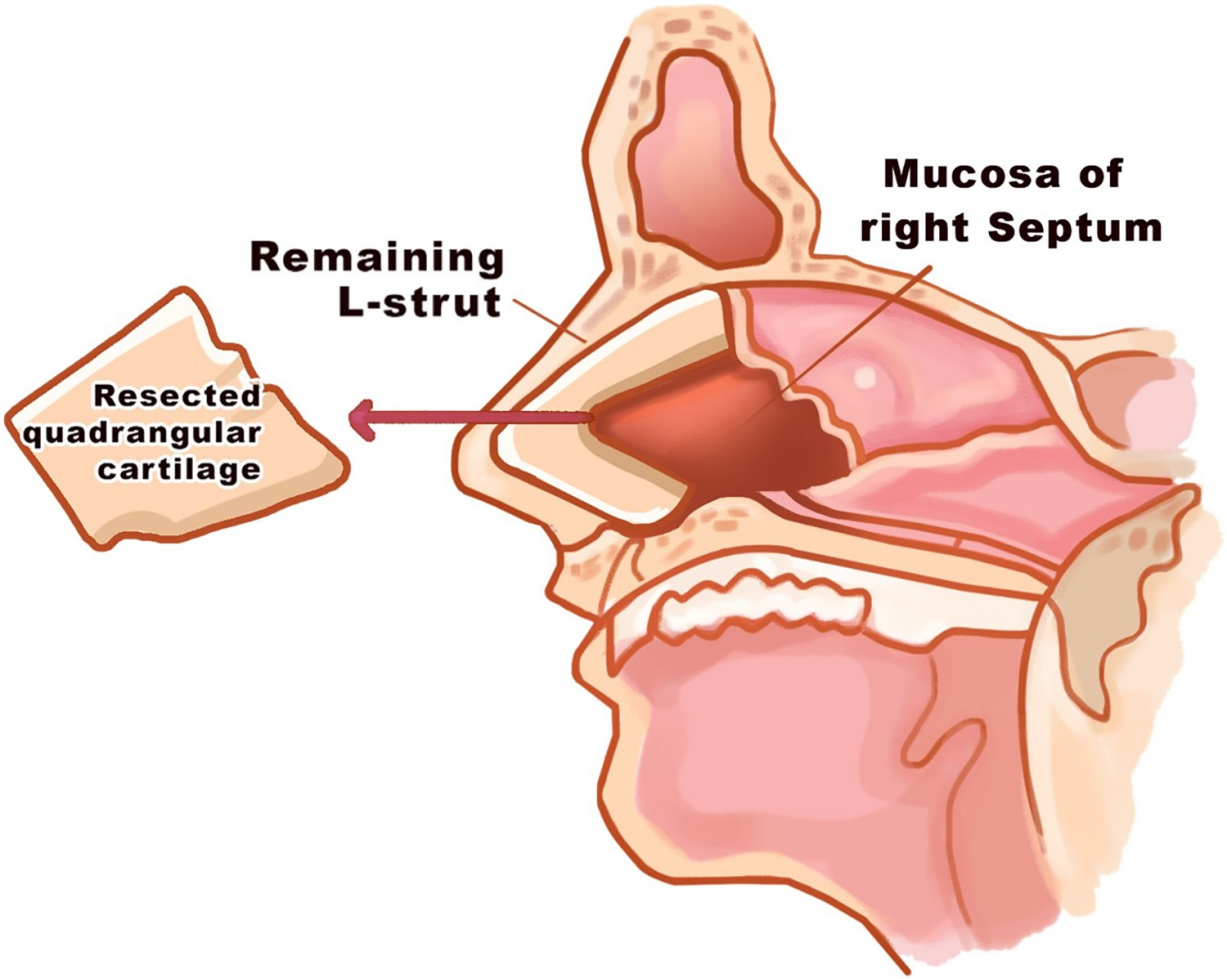
Illustration of residual dimension of septal L-strut.

**Fig 3:**
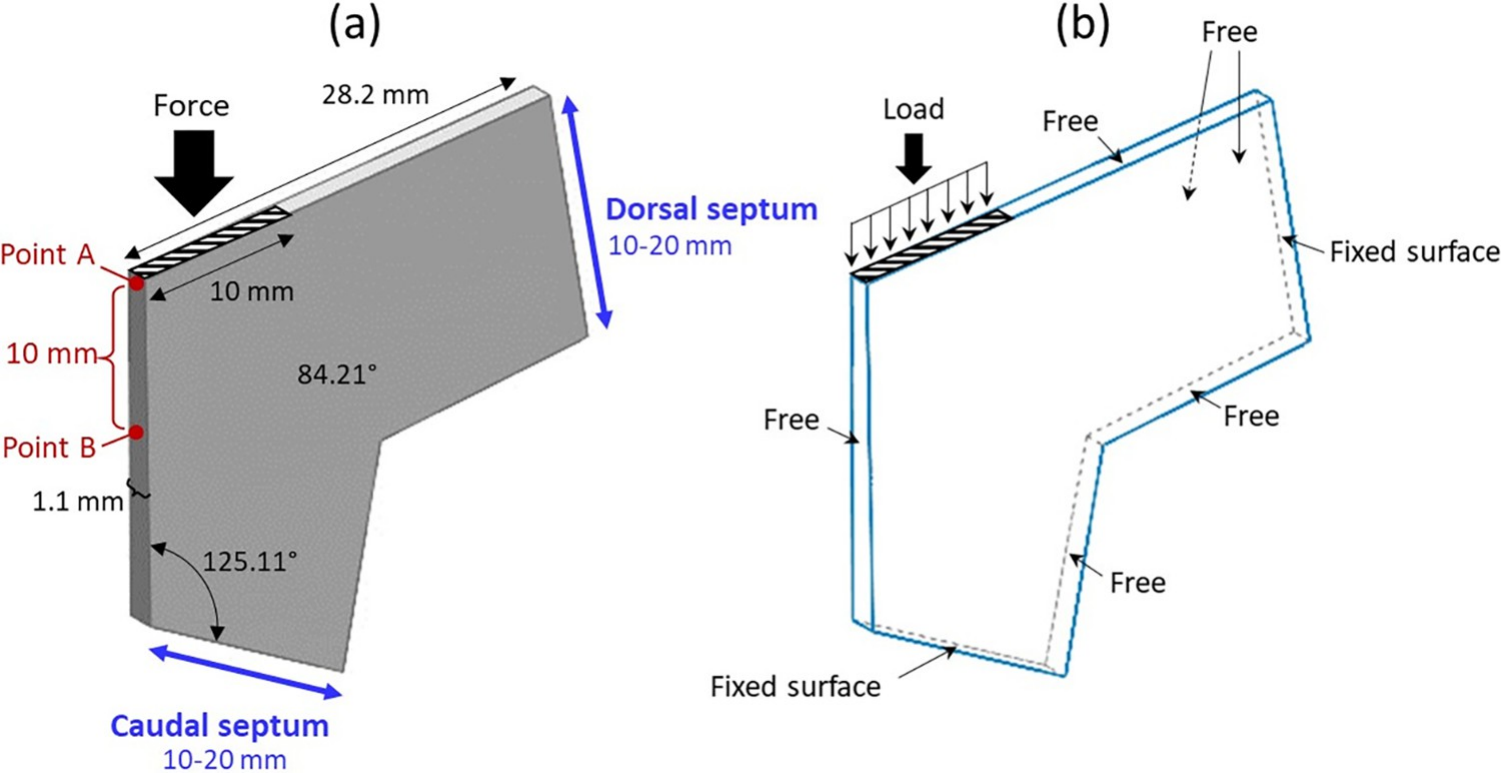
(a) the model of septal L-strut design with dimensions and measurement points. (adapted from Lee et al., 2015 [24]) (b) the boundary conditions for mechanical deformation analysis.

The illustration of residual of the septal L-strut structure and its position are presented in Fig 2. The model in this study is anatomically created as appears in Fig 3 as well as its dimensions. The strength of the septal L-strut was analyzed at the widths of dorsal septum and caudal septum within the range of 10–20 mm. The dimensions and thickness of the model are adapted from Lee et al., 2015 [24]. The maximum load that the septal cartilage can tolerate is applied on the dorsal surface of septal L-strut as seen in Fig 3(A).

### Mechanical deformation analysis

The concept of equations in mechanics is applied to describe a deformation behavior in this study. The equations of mechanical deformation [28] show relation between stress, strain and displacement. The stresses occur when the force is applied to a tissue. This can be explained by equilibrium equations (Eq (1)). The value of stress for a known value of the strain, demonstrates as constitutive relation (Eq (2)). Then compatibility equation (Eq (3)) places restrictions on how the strains can vary over the body so that displacement field could be found for the assumed strain field, as described in strain-displacement relation (Eq (4)) as follows:

$$\frac{\partial \sigma_{xx}}{\partial x}+\frac{\partial \sigma_{xy}}{\partial y}+\frac{{\partial \sigma }_{xz}}{\partial z}=0$$

$$\frac{\partial \sigma_{xy}}{\partial x}+\frac{\partial \sigma_{yy}}{\partial y}+\frac{{\partial \sigma }_{yz}}{\partial z}+F_{y}=0$$ (1)

$$\frac{\partial \sigma_{xz}}{\partial x}+\frac{\partial \sigma_{yz}}{\partial y}+\frac{\partial \sigma_{zz}}{\partial z}=0$$

Where *F* = *ma*, F is force applied on tissue (N), *m* is mass (kg), *g* is the gravitational force (N) and *μ* is coefficient of friction. In this study, the maximally applied load that septal L-strut can tolerate before deformation for each dimension is measured.

$$\sigma_{xx}=\frac{1}{E}\left[\varepsilon_{xx}-\nu (\varepsilon_{yy}+\varepsilon_{zz})\right]$$

$$\sigma_{yy}=\frac{1}{E}\left[\varepsilon_{yy}-\nu (\varepsilon_{xx}+\varepsilon_{zz})\right]$$ (2)

$$\sigma_{zz}=\frac{1}{E}\left[\xi_{zz}-\nu (\varepsilon_{xx}+\varepsilon_{yy})\right]$$

$$\sigma_{xy}=\varepsilon_{xy}\left(1+\nu \right)/E$$

$$\sigma_{xz}=\varepsilon_{xz}\left(1+\nu \right)/E$$

$$\sigma_{yz}=\varepsilon_{yz}\left(1+\nu \right)/E$$

$$\frac{\partial^{2}\varepsilon_{xx}}{\partial y^{2}}+\frac{\partial^{2}\varepsilon_{yy}}{\partial x^{2}}=\frac{\partial^{2}\gamma_{xy}}{\partial x\partial y}$$

$$\frac{\partial^{2}\varepsilon_{zz}}{\partial y^{2}}+\frac{\partial^{2}\varepsilon_{yy}}{\partial x^{2}}=\frac{\partial^{2}\gamma_{yz}}{\partial y\partial z}$$

$$\frac{\partial^{2}\varepsilon_{xx}}{\partial y^{2}}+\frac{\partial^{2}\varepsilon_{zz}}{\partial x^{2}}=\frac{\partial^{2}\gamma_{zx}}{\partial z\partial x}$$ (3)

$$\frac{\partial }{\partial x}\left(\frac{\partial u_{zx}}{\partial y}+\frac{\partial u_{xy}}{\partial z}-\frac{\partial \gamma_{zy}}{\partial x}\right)=\frac{2\partial^{2}\varepsilon_{xx}}{\partial y\partial z}$$

$$\frac{\partial }{\partial y}\left(\frac{\partial u_{xy}}{\partial z}+\frac{\partial u_{zy}}{\partial x}-\frac{\partial \gamma_{zx}}{\partial x}\right)=\frac{2\partial^{2}\varepsilon_{yy}}{\partial z\partial x}$$

$$\frac{\partial }{\partial z}\left(\frac{\partial u_{zy}}{\partial x}+\frac{\partial u_{zx}}{\partial y}-\frac{\partial \gamma_{xy}}{\partial z}\right)=\frac{2\partial^{2}\varepsilon_{zz}}{\partial x\partial y}$$

$$\varepsilon_{xx}=\frac{\partial \gamma_{x}}{\partial x},\varepsilon_{yy}=\frac{\partial \gamma_{y}}{\partial y},\varepsilon_{zz}=\frac{\partial \gamma_{z}}{\partial z}$$

$$\varepsilon_{xy}=\frac{1}{2}\left(\frac{\partial \gamma_{x}}{\partial y}+\frac{\partial \gamma_{y}}{\partial x}\right)$$ (4)

$$\varepsilon_{xz}=\frac{1}{2}\left(\frac{\partial \gamma_{x}}{\partial z}+\frac{\partial \gamma_{z}}{\partial x}\right)$$

$$\varepsilon_{yz}=\frac{1}{2}\left(\frac{\partial \gamma_{y}}{\partial z}+\frac{\partial \gamma_{z}}{\partial y}\right)$$

Young’s modulus, Poisson’s ratio and shear modulus relationship for isotropic and homogeneous materials are as follow;

$$G=\frac{E}{2(1+\nu )}$$ (5)

where *σ* denotes the stress (Pa), *ε* is the mechanical strain, *E* is Young’s modulus (Pa), *G* is shear modulus (Pa), *ν* is the Poisson’s ratio and *γ* is the average displacement (m).

To simplify the problem, the following assumptions are made:

1. Three-dimensional model with the Cartesian coordinate system is assumed.
2. Septal cartilage is assumed to be homogeneous and isotropic incompressible linearized elastic material.

### Boundary conditions

The boundary conditions of mechanical deformation analysis are indicated in Fig 3(B). A uniform vertical loading force is applied on the horizontal surface of the nasal tip. The caudal and dorsal osteocartilaginous junctions are constrained as fixed surface. The rest of the surfaces are set at free boundary condition.

## Verification of the model

In order to verify the accuracy of the numerical model in this work, the septal L-strut model is validated against the experimental results performed by Department of Anatomy, Faculty of Medicine, Siriraj Hospital, Mahidol University, Thailand. The process of verification included testing of the warping tolerance of the nasal septum at the different dimensions of septal L-strut in fresh frozen human cadavers. There was no report regarding the patient data in this study. This part was approved by the Ethics Committee of the Faculty of Medicine Siriraj Hospital, Mahidol University, Thailand, protocol number 1012/2564.

**Fig 4:**
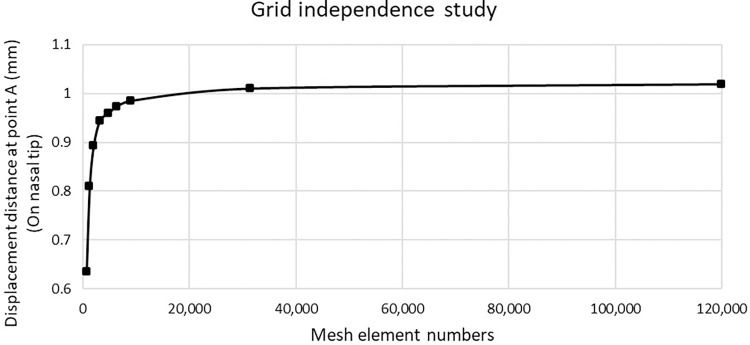
Grid independence study.

The maximum forces exerted on nasal tip without causing deformity are compared with the same conditions. The displacement distances of different dimension of septal L-strut (dorsal septum x caudal septum) are evidentially paired between computational and experimental results, as can be seen in Table 2. Thus, the reliability of the model in this study is proofed and can be used for performing an accurate prediction. Furthermore, grid independence study is shown in Fig 4. The mesh element number of 120,000 is the most suitable approximation for this simulation.

**Table 2:** The result validation of the septal L-strut model.

Residual (Dorsal x Caudal)	Displacement distance at point A (On nasal tip)	
	Experimental results	Computational results
10 x 10 mm	1 mm	1.01963 mm
12 x 12 mm	1 mm	1.00301 mm
14 x 14 mm	1 mm	1.02277 mm
16 x 16 mm	1 mm	1.03303 mm

## Results

Three-dimensional models of the septal L-strut with multiple geometries were investigated in this study. The models were subjected to a loading force applied to the nasal tip surface. Mechanical deformation analysis was performed to calculate the Von Mises stress and total elastic strain energy. The study examined the influence of dimensions, cutting angles, and fillet angles on force tolerance and structural integrity. Based on the findings, recommended designs for the septal L-strut and a new design for the septal strut in septoplasty procedures were proposed to improve surgical outcomes.

**Fig 5:**
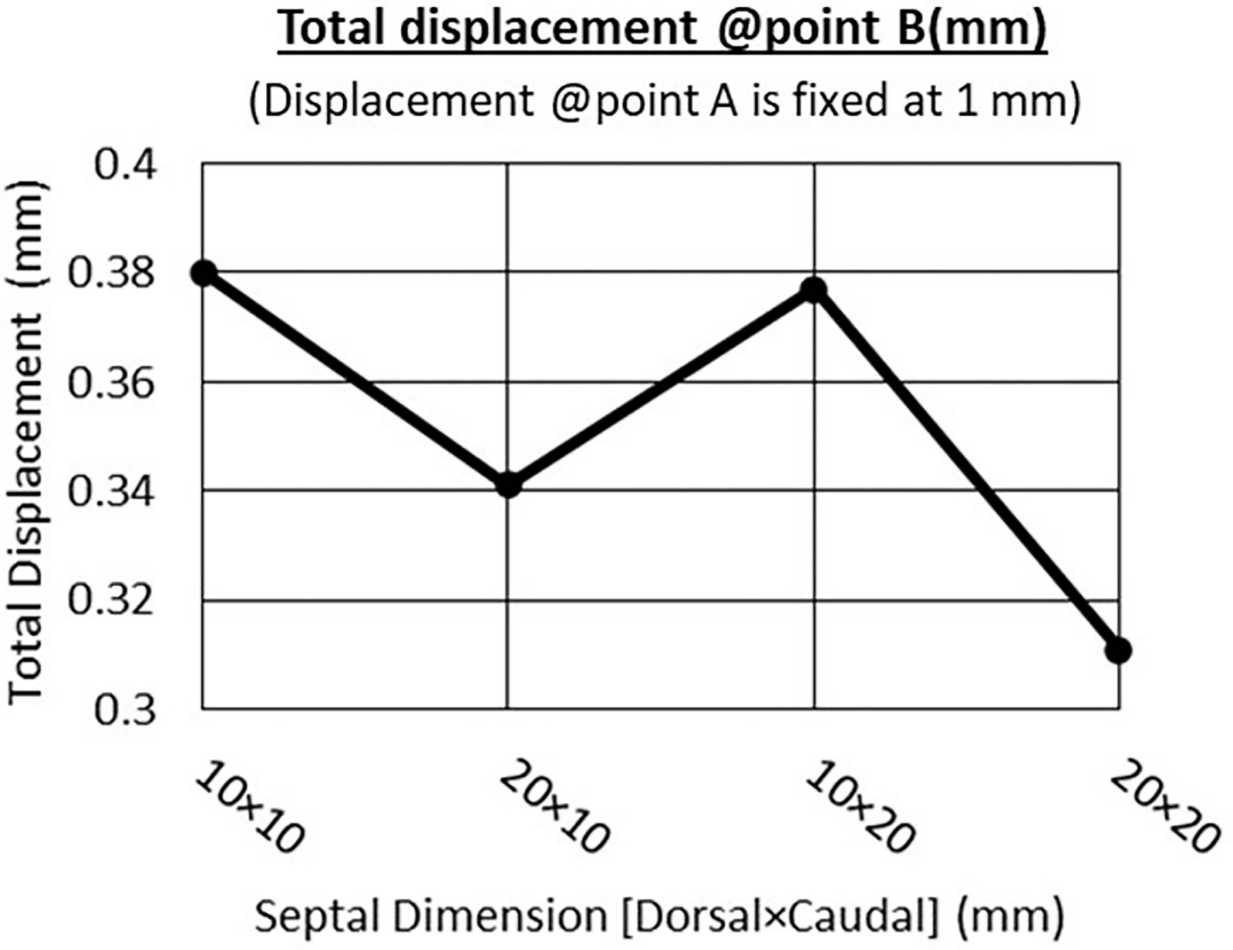
Total displacement at point B (mm), vary with different dimensions, displacement at point A is fixed at 1 mm.

**Fig 6:**
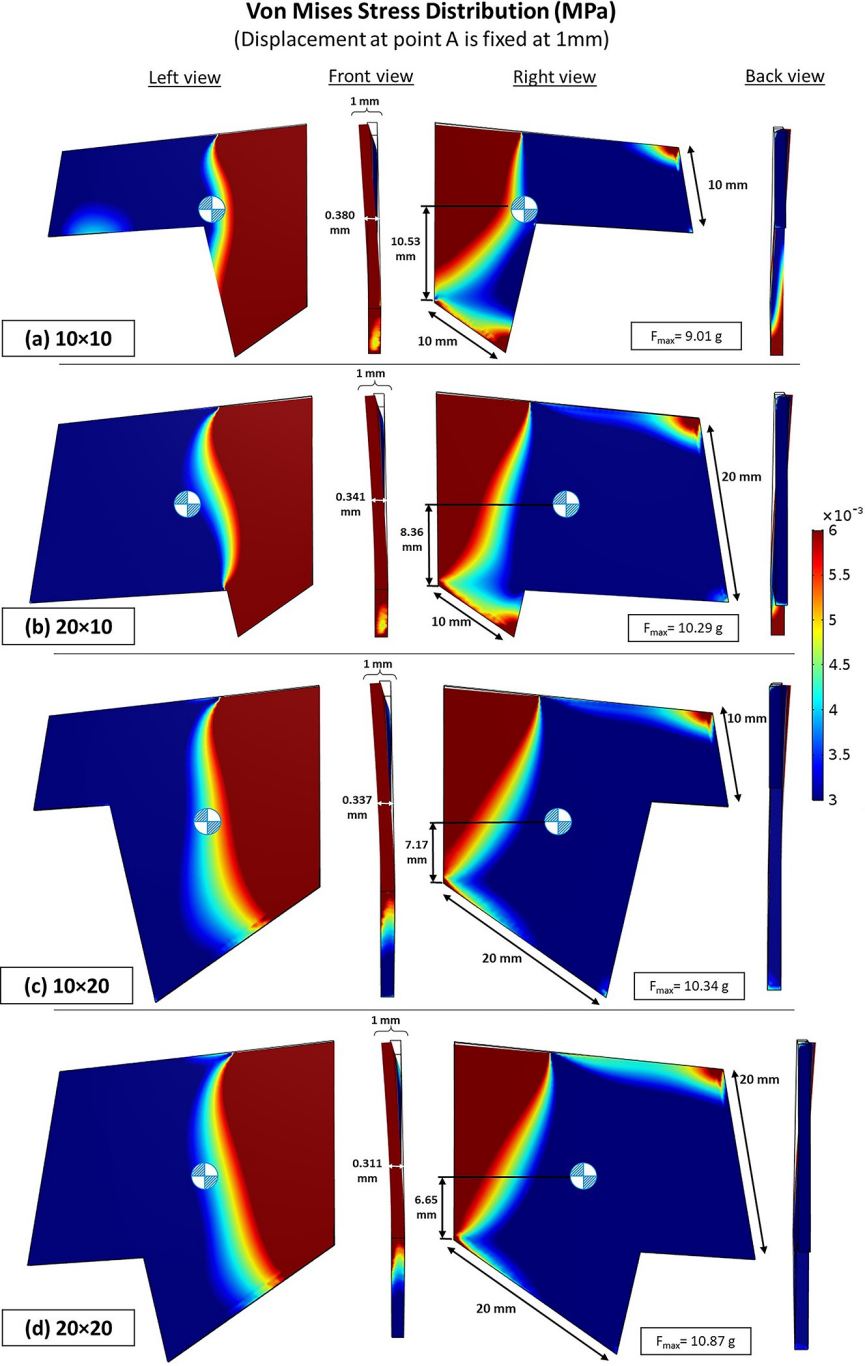
Von Mises stress distribution of septal L-strut model (MPa), vary with different dimensions, displacement at point A is fixed at 1 mm.

The aim of this study is to analyze the effects of different dimensions of the septal L-strut on its deformation characteristics and supporting ability. When comparing cases with the same septal tip displacement, it was observed that the deformation at point B varied, despite having the same displacement distance at the tip (Figs 5 and 6). The case with dimensions of 20 x 20 mm exhibited the highest core strength and stability, supporting the maximum loading force and displaying the least deformity at point B. Increasing the width of either the dorsal or caudal septum improved the strength of the septal L-strut. Specifically, a wider caudal septum dimension (Fig 6(C)) showed greater force tolerance compared to a wider dorsal septum dimension (Fig 6(B)). The model with dimensions of 20 x 20 mm demonstrated both lower core destruction (Fig 6(D)) and the lowest center of gravity compared to the other models.

**Fig 7:**
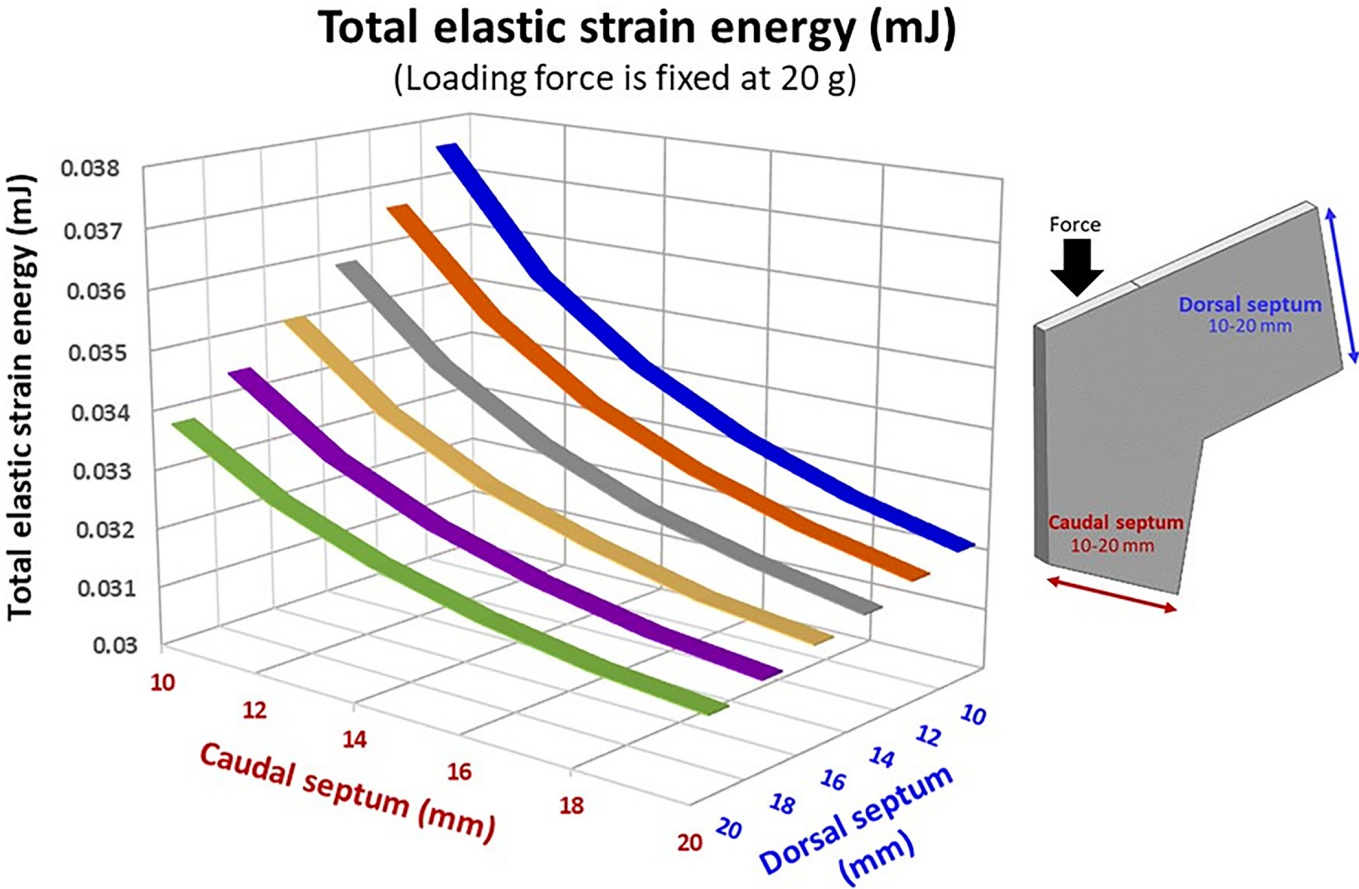
Total elastic strain energy (mJ), vary with different dimensions (dorsal and caudal septum), loading force is fixed at 20 g.

**Fig 8:**
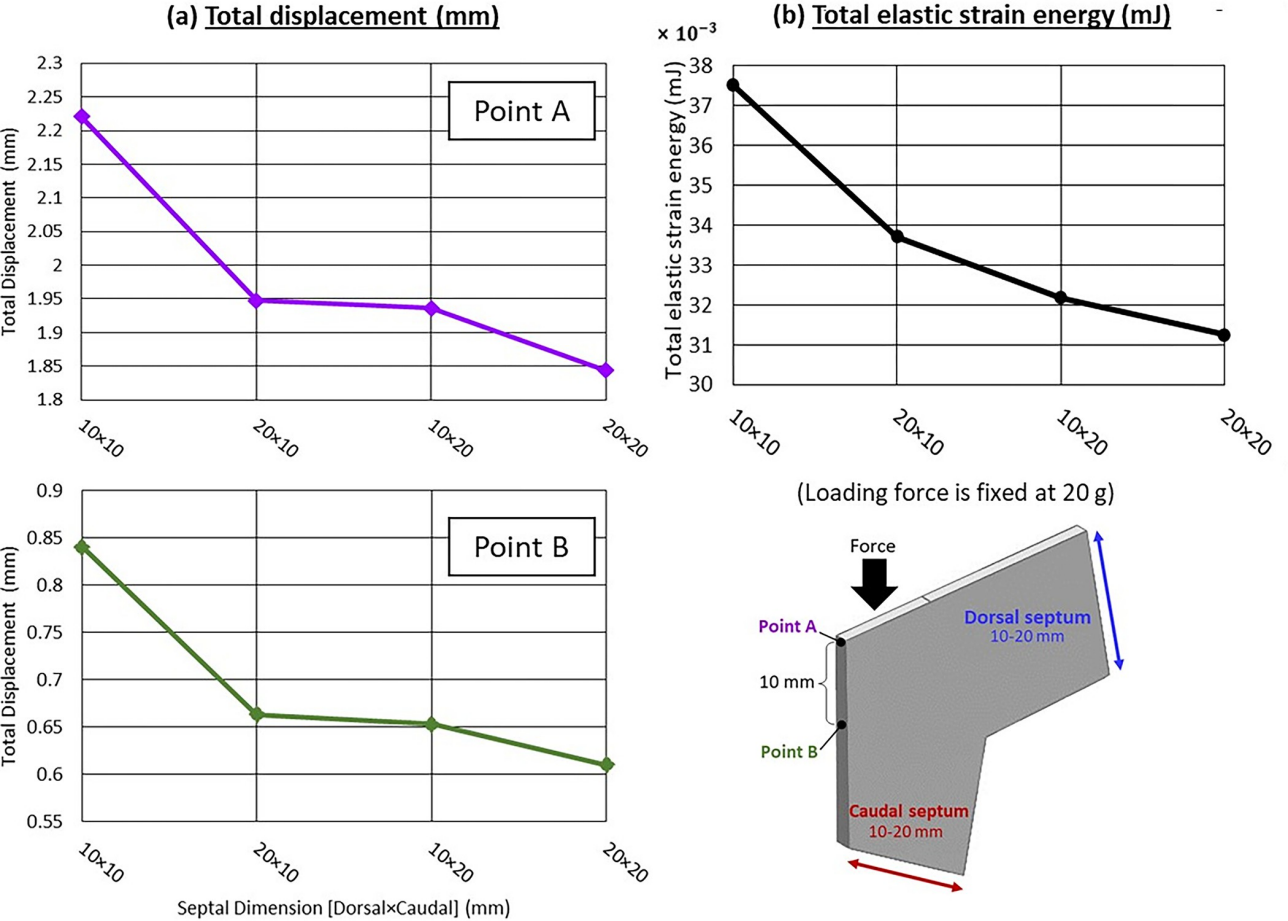
(a) Total displacement (mm) (b) Total elastic strain energy (mJ), vary with different dimensions (dorsal and caudal septum), loading force is fixed at 20 g.

Under a loading force of 20 g, the widths of the dorsal and caudal septum were found to influence the supporting ability of the septal L-strut. Decreasing the width of either side resulted in increased elastic strain energy and deformation, while wider widths provided better support with reduced strain (Fig 7). Fig 8 indicated that a wider caudal septum (10 x 20 mm) caused less displacement at both points A and B compared to a wider dorsal septum (20 x 10 mm). The model with dimensions of 20 x 20 mm exhibited the strongest support, minimizing the deformity distance at both points A and B.

**Fig 9:**
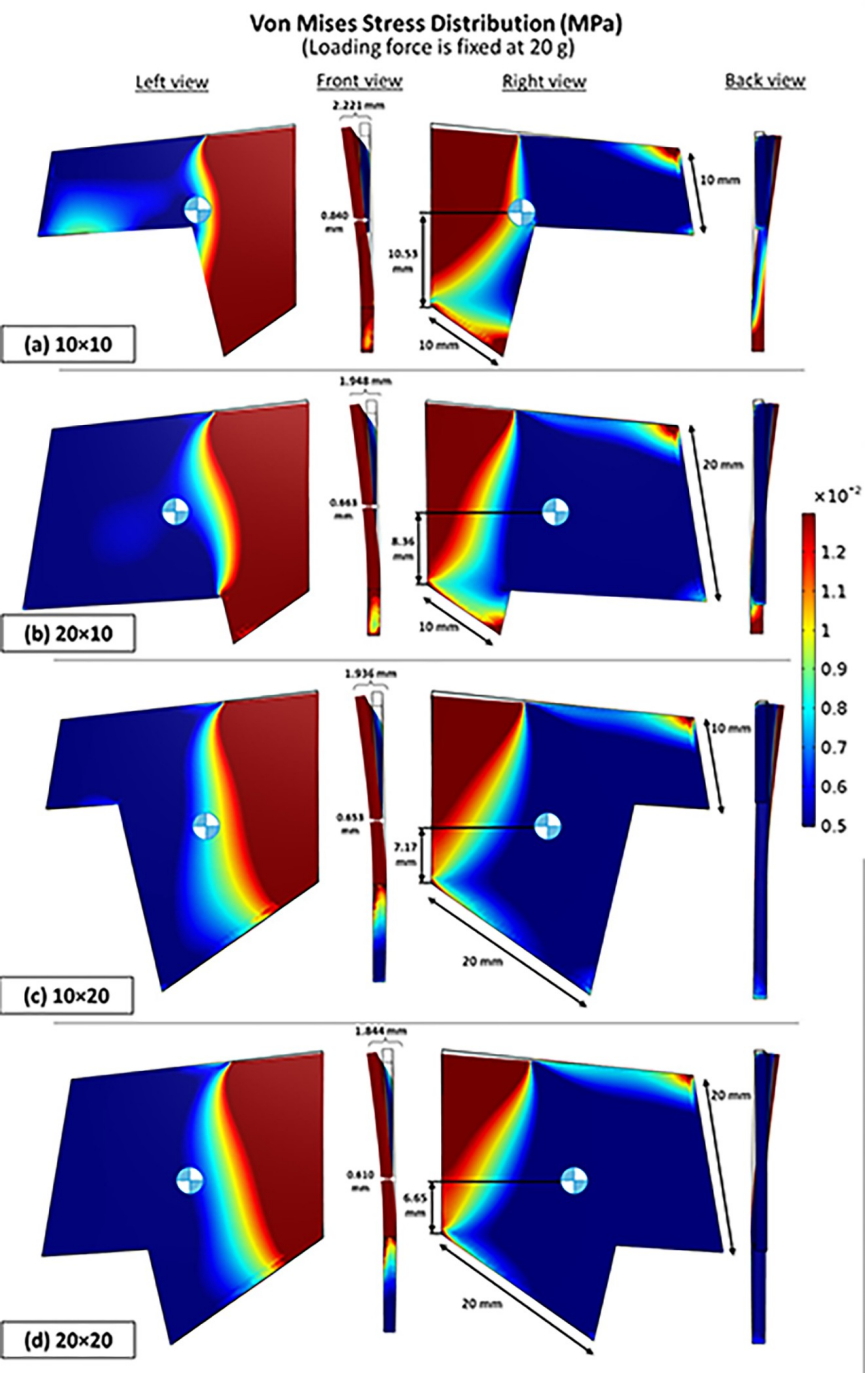
Von Mises stress distribution (MPa), vary with different dimensions (dorsal × caudal septum) (a) 10 x 10 mm (b) 20 x 10 mm (c) 10 x 20 mm and (d) 20 x 20 mm, loading force is fixed at 20 g.

The von Mises stress distribution demonstrated that different dimensions of the septal L-strut exhibited varying warping tolerance (Fig 9). The models with dimensions of 10 x 10 mm, 20 x 10 mm, 10 x 20 mm, and 20 x 20 mm showed increasing strength from weakest to strongest, respectively. The stronger models displayed less concentrated stress in the lower septal area, closer to the basement, and had lower centers of gravity.

**Fig 10:**
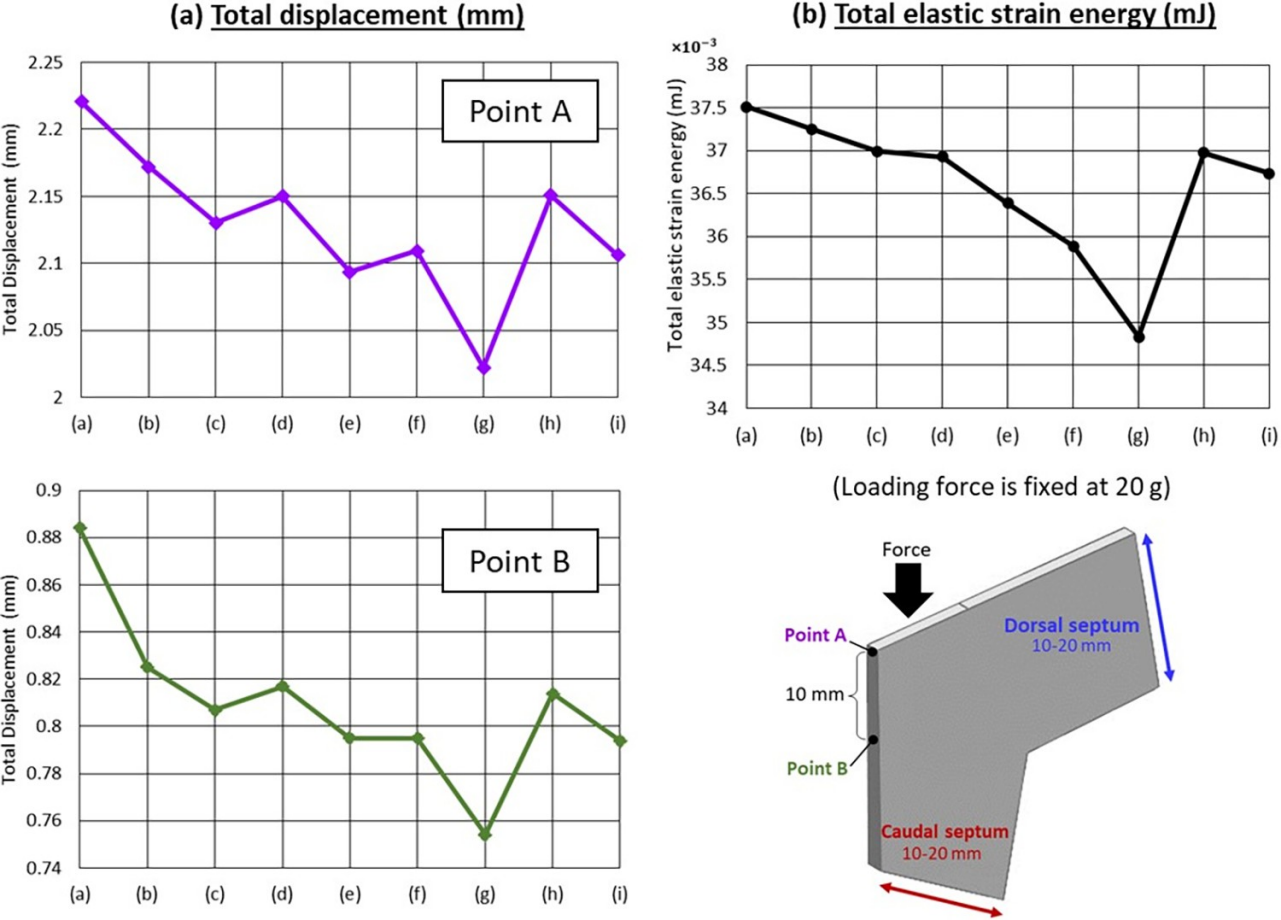
(a) Total displacement (mm) (b) Total elastic strain energy (mJ), vary with different cutting angles (see in Fig 12), loading force is fixed at 20 g.

**Fig 11:**
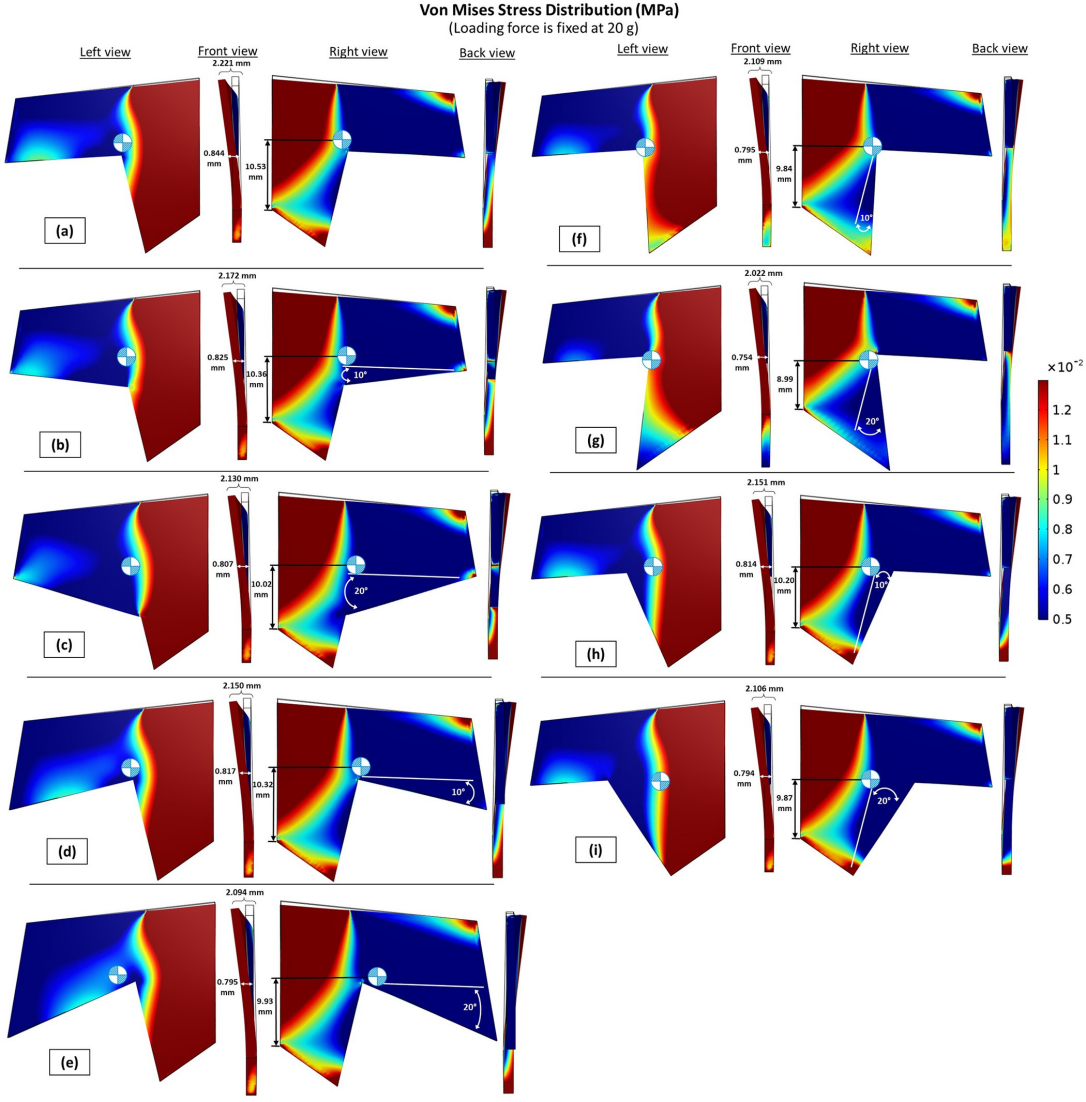
Von Mises stress distribution of models with different cutting angle, loading force is fixed at 20 g.

The cutting angle, defined as the angle between the cutting face of the new shape and the surface of the standard shape of the septal L-strut, had a significant impact on its strength and stability (Figs 10 and 11). Adjusting the cutting angle resulted in improved outcomes, including reduced strain and displacement. Among the different models, Fig 10(G) exhibited the highest structural integrity, displaying the least displacement and strain energy. This observation can be further understood by examining the von Mises stress distribution (Fig 11). The wider support provided by the base area minimized stress near the caudal septum region (Fig 11(G)). Consequently, a wider structural basement, specifically the caudal septum, resulted in a lower center of gravity, as a wider base offers greater strength and stability.

**Fig 12:**
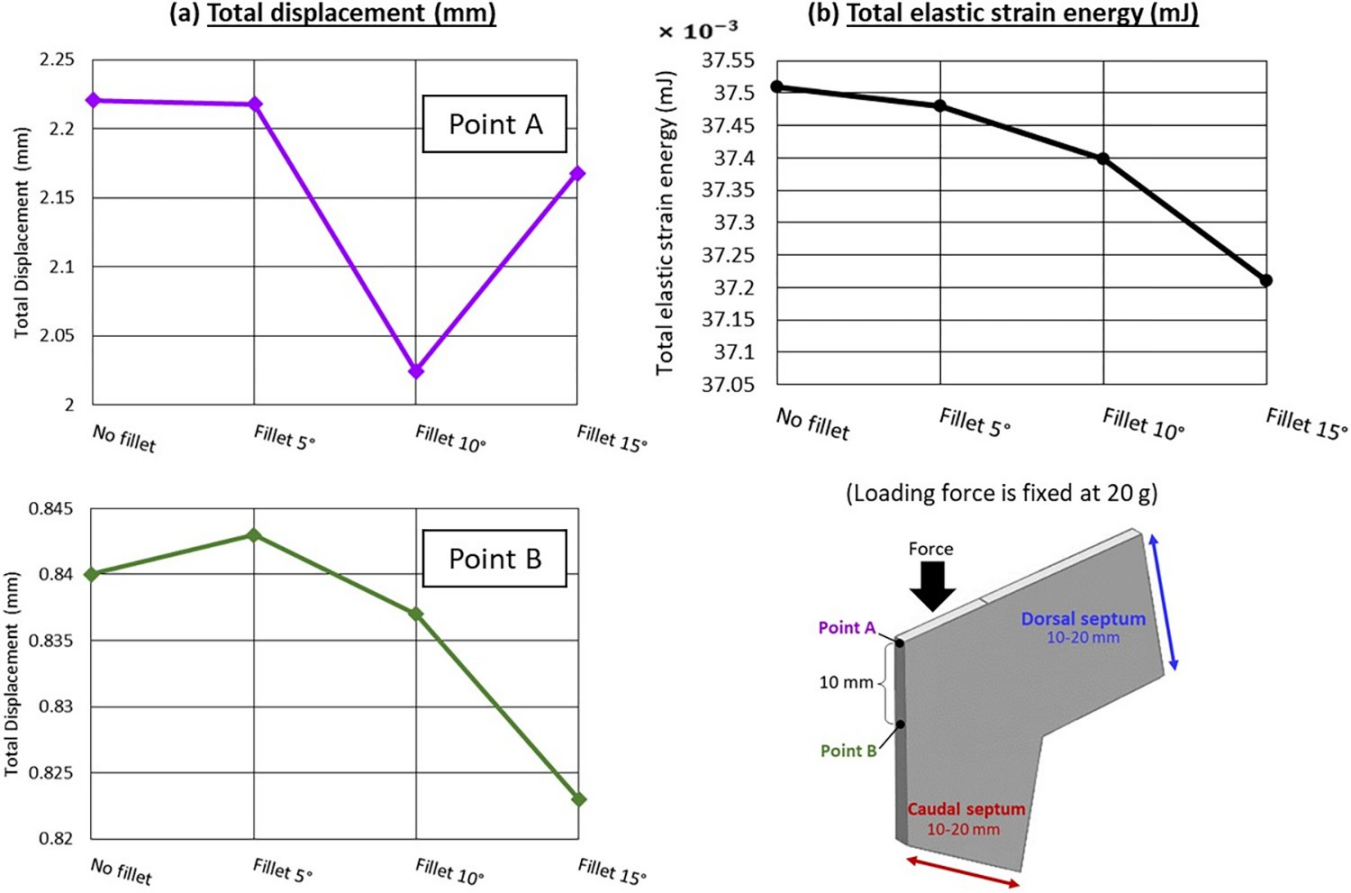
(a) Total displacement (mm) (b) Total elastic strain energy (mJ), vary with different fillet radius, loading force is fixed at 20 g.

**Fig 13:**
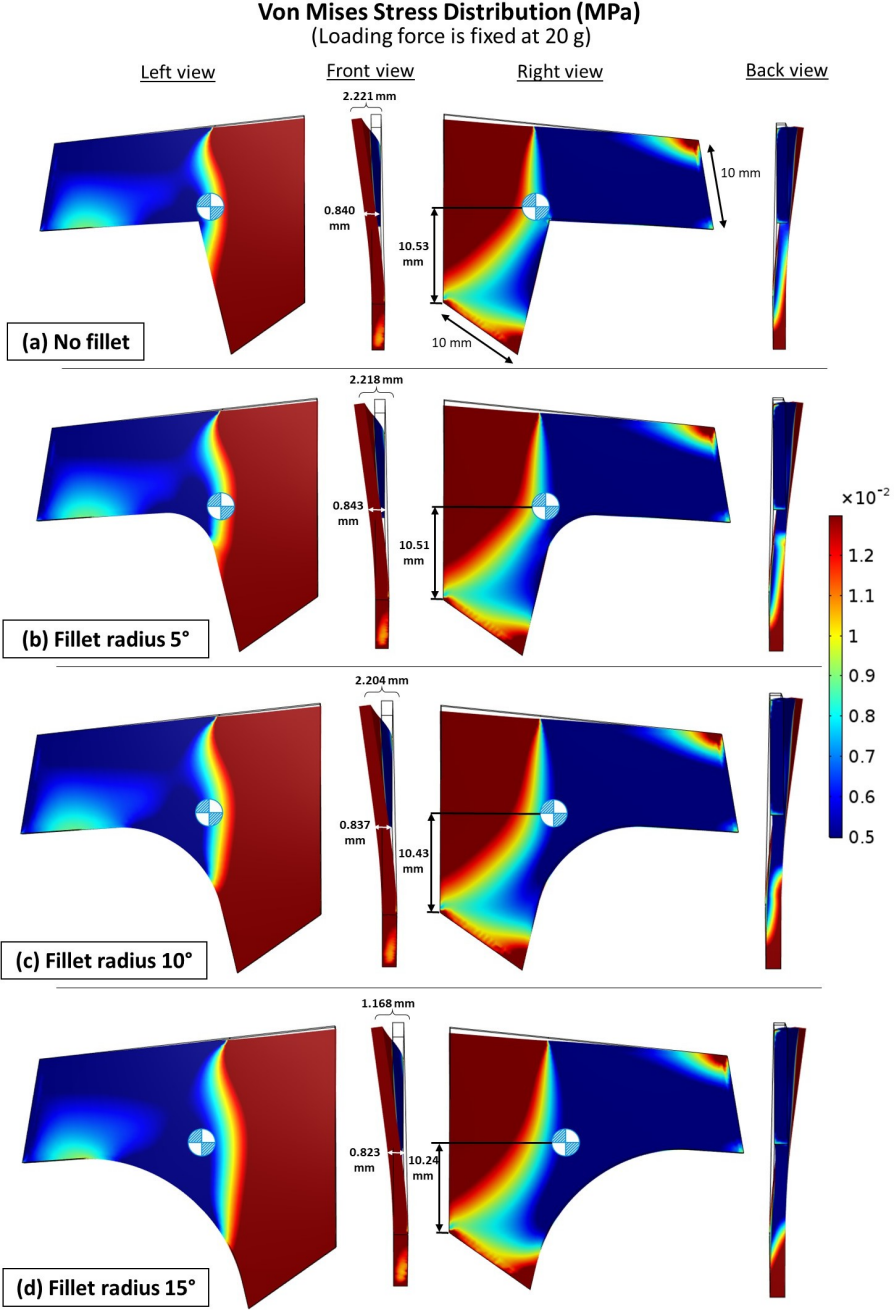
Von Mises stress distribution of models with different fillet radius, loading force is fixed at 20 g.

A fillet angle refers to the angle formed between two intersecting surfaces or edges, resulting in rounded corners instead of sharp ones. The size of the fillet angle determines the degree of curvature at the corner. The study findings indicate that fillet angles outperformed sharp angles in terms of enhancing the durability of the septal L-strut. Among the different models, the one with a fillet radius of 15° (Fig 12) exhibited the lowest amount of total elastic strain energy compared to 5°, 10°, and sharp edges. The displacement at point B was directly influenced by the amount of strain, with smaller displacements indicating less deformation. This sensitivity is due to point B’s proximity to the structural core. Furthermore, greater interior corner reduced stress at the corner and limited its transfer to the middle part of the septal L-strut. This shift in stress distribution lowered the location of the gravitational center (Fig 13).

**Fig 14:**
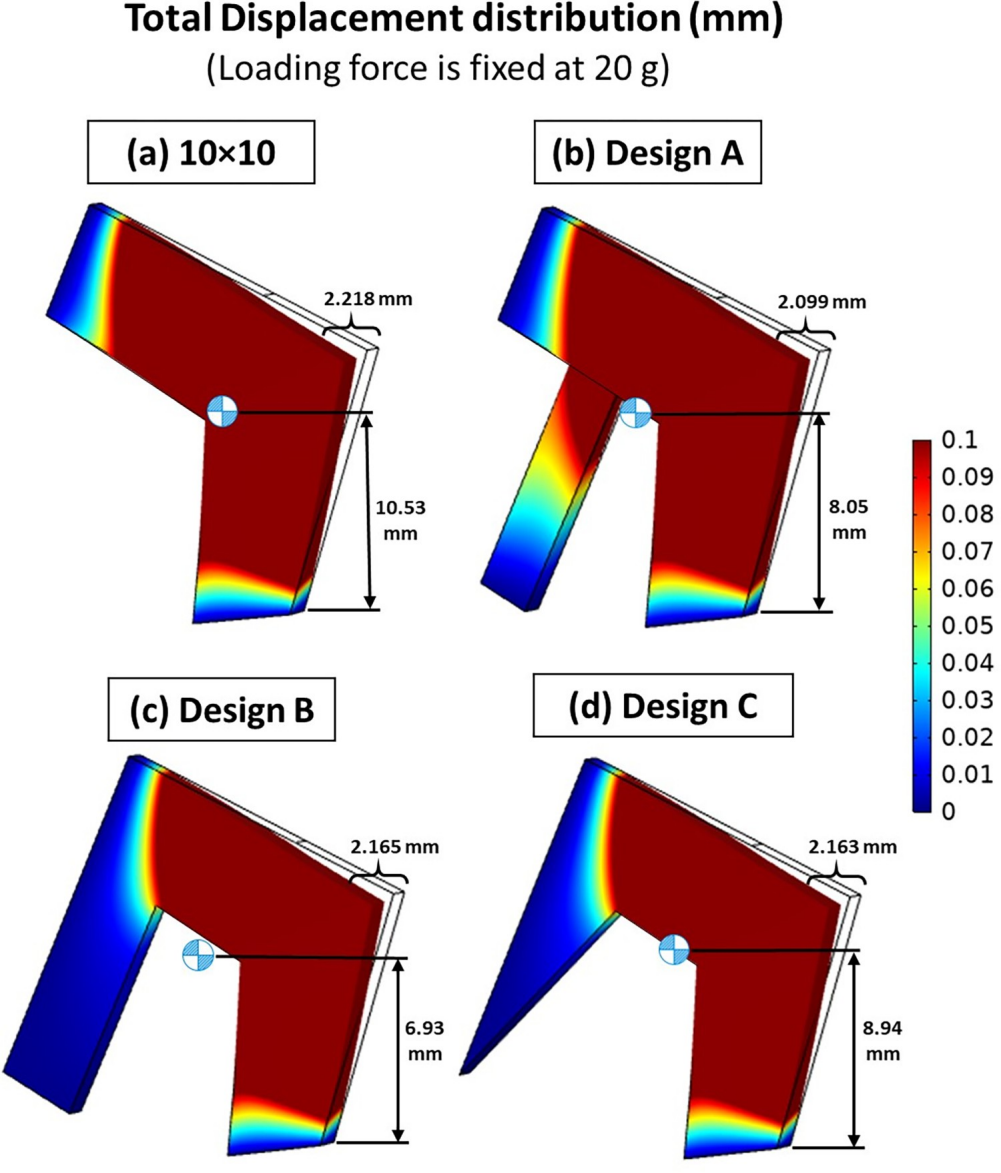
The comparison of total displacement between standard and new techniques, under loading force of 20 g (a) standard technique of 10 x 10 mm (b) design A: septal support graft (invented technique in this study) (c) design B: straight C-shaped [17] (d) design C: slope C-shaped [17].

In septoplasty procedures, there is no standardized approach for the septal L-strut technique, with variations depending on the surgeon’s experience and individual patient characteristics. However, one reliable method involves using a septal extension graft (SEG) to reinforce the nasal septum, aiming to control tip projection and rotation after surgery [18, 19, 29]. The SEG involves using cartilage grafts as buttresses, combined with the nasal septum, to reinforce the nasal cartilage structure [30]. In this study, we propose a novel technique called the "septal support graft" (design A), inspired by the SEG concept, strategically placed at the center of gravity. The results demonstrate that this method offers improved load-bearing capacity and stability when compared to using the L-strut alone (Fig 14(A)).

The study explored alternative designs based on a previously developed technique in a clinical study. These designs, referred to as C-shaped septal struts (design B and C, Fig 14(C) and 14(D)), incorporate two vertical limbs on both ends, which have been previously proposed in clinical studies without computational evidence [17]. The computational analysis showed that the C-shaped septal struts and septal support graft had better strength than the standard method. However, among all the designs, the septal support graft (design A) exhibited the highest strength compared to C-shaped (design B and C). Interestingly, the gravitational center was found to be lowest for design B, followed by design A and design C, respectively.

## Discussions

According to mechanical engineering theory, when a load is applied to septal cartilage, it generates stresses that result in deformation due to strains. The likelihood of failure is influenced by material properties such as Young’s modulus, Poisson’s ratio, and density. Additionally, geometric properties such as length, width, thickness, shape, and corner angles contribute to stress concentration, playing a crucial role in determining structural strength.

In relation to the stress equation ($\sigma =\frac{F}{A}$), expanding the area of septal support, particularly the caudal septum surface, has shown promising results in enhancing structural integrity. By increasing the surface area, the distribution of stress becomes more efficient, resulting in reduced strain. This explains why a wider caudal septum provides improved stability and can better withstand external forces, as the pressure per unit area is minimized. Notably, this finding aligns with the established principle that the caudal septum serves as a crucial support for the nasal tip [31]. Additionally, widening the caudal septum has the added benefit of lowering the center of gravity. Consequently, a broader caudal septum, serving as a solid foundation, significantly contributes to the overall strength of the septal L-strut. Thus, the evidence strongly suggests that the width of the caudal septum carries more influence compared to that of the dorsal septum.

### The effect of dorsal septum and caudal septum dimensions

The study discovered that varying the dimensions of the dorsal and caudal septum of the septal L-strut can result in similar septal tip displacement but different deformation characteristics. Widening the caudal septum dimension was found to offer stronger support compared to widening the dorsal septum dimension. Therefore, it is important to consider the dimensions of the septal L-strut in order to enhance support and stability during surgical procedures.

### The effect of cutting angle

The cutting angle of the septal L-strut plays a significant role in determining its strength and stability. Surgeons can manipulate this angle to achieve improved results by minimizing strain and deformation. An interesting finding from the study is that when the cutting angle induces a wider caudal septum, it results in increased strength and a lower center of gravity. By considering and adjusting the cutting angle to create a wider caudal septum, surgeons can enhance the structural integrity and stability of the septal L-strut.

### The effect of fillet angle

The shape of the fillet angle at the interior corner of the septal L-strut is important for improving its durability. Rounded corners created by fillet angles perform better performance than sharp corners. In particular, the model with a larger fillet angle showed the least amount of elastic strain energy, indicating less deformation and better structural integrity. Additionally, increasing the fillet radius at the interior corner reduces stress concentration in that area, which prevents damage to the central part of the strut. Furthermore, a larger fillet angle has the added benefit of shifting the center of gravity lower, which contributes to improved stability and overall performance of the septal L-strut.

### The computational performing of new techniques

This study examines the importance of using structural support techniques, such as the septal support graft and C-shaped septal struts, in septoplasty procedures. These techniques significantly improve stability by optimizing the load-bearing capacity. Among these techniques, the septal support graft demonstrates the highest strength and stability due to its superior load-bearing capacity. Interestingly, despite having a higher center of gravity, the septal support graft remains exceptionally stronger and more stable compared to the C-shaped technique. This can be attributed to other critical factors, such as the distribution of mass, beam design, and unique characteristics of the structure.

### The suggestions to improve integrity and prevent structural failure

To enhance the integrity of the septal L-strut and achieve better surgical outcomes, we propose the following recommendations, drawing from the fields of mechanical engineering and plastic surgical methodology:

1. Expand the structural basement (caudal septum): By widening the caudal septum, the center of gravity will shift lower, resulting in improved strength and stability. This adjustment also allows the center of gravity to restore itself more effectively to its original position after temporary deformation, contributing to better overall performance.
2. Modify the geometry: One effective approach is to transform the corner edges into fillet shapes. This alteration also helps to shift the center of gravity to a lower position. As a result, the durability and stability of the septal L-strut can be significantly enhanced.
3. Consider the impact of loading force and septal geometry: The distribution of stress, strain, and deformation within the L-strut is influenced by the location of the loading force and the specific septal geometry. Therefore, relocating the loading force can lead to different outcomes, highlighting the need for careful consideration and analysis.

**Fig 15:**
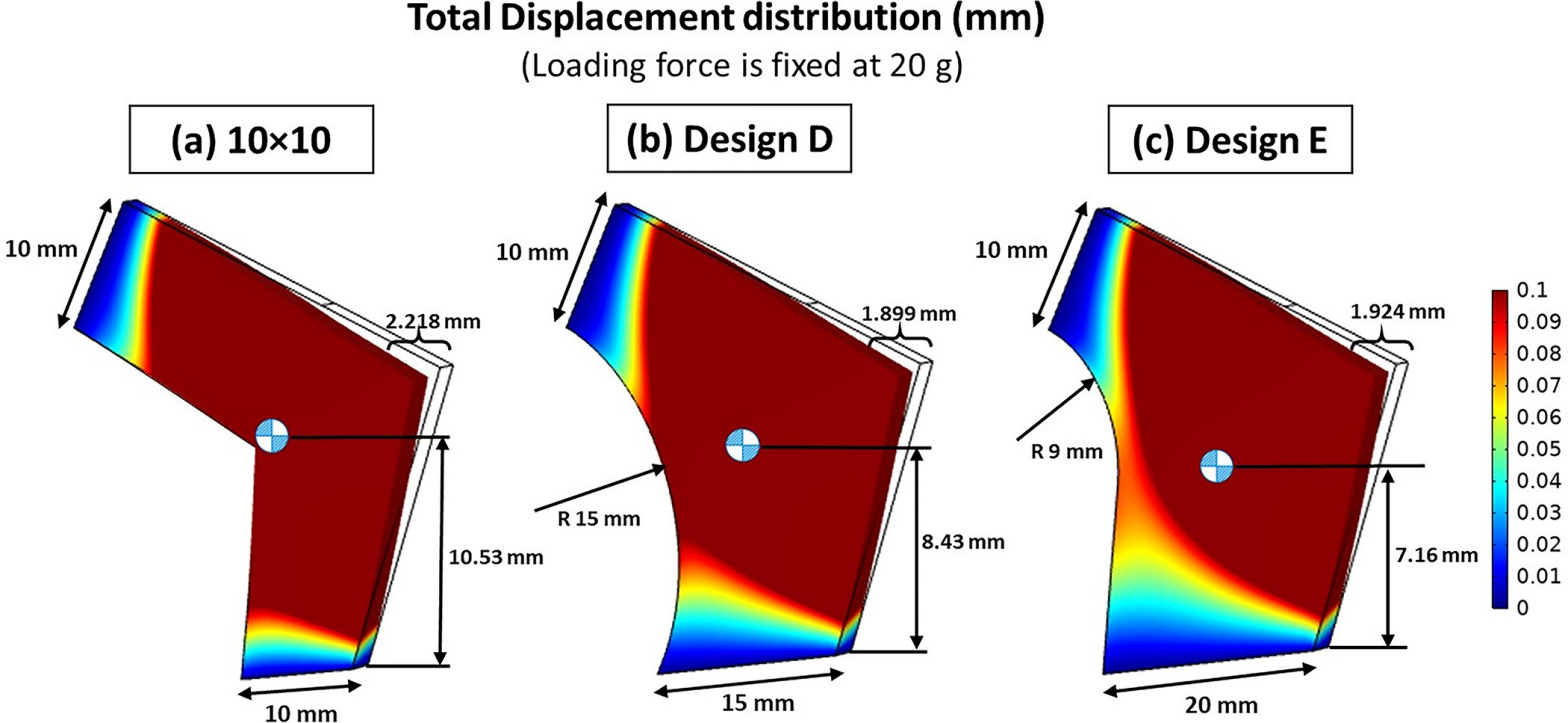
The comparison of total displacement between standard and invented techniques in this study, under loading force of 20 g (a) standard technique of 10 x 10 mm (b) and (c) invented techniques in this study.

Based on the findings of this study, we propose design modifications for the septal L-strut to enhance its strength and durability. Specifically, we recommend increasing the width of the basement and reshaping the interior corners from sharp angles to fillet angles. In our comparative analysis, Design D and E (Fig 15(B) and 15(C)) demonstrated better outcomes, with reduced deviation at the nasal tip compared to the 10x10 mm case (Fig 15(A)). Notably, our study revealed that the radius of the fillet corner has a greater impact on the structural stability of the L-strut than the width of the basement.

### Limitations

It is important to note that the findings of this study are derived from a computational model based on the concept of medical engineering, with a specific focus on the nasal septum structure. Thus, there are limitations in this study to consider. Firstly, the nasal tip support, in fact, is not exclusively from nasal septum, but rather from contributions of many adjacent structures [31]. Secondly, the verification model of the Finite Element Analysis was performed by the data in Asian Cadavers [23] that may limit its accuracy in the population with a different ethnicity. Thirdly, accurate finite element analysis relies on essential model validation due to the need for simplifying complex models during simulation. Lastly, the newly developed models have not been undergone testing on actual patients. Therefore, further clinical studies should be conducted to validate the practical application of these design concepts.

## Conclusions

This study aimed to optimize the strength and stability of rhinoplasty procedures by analyzing the geometric properties and gravitational center of the septal L-strut. Our findings suggest that widening the caudal septum and preserving its inherent strength through the use of rectangular-cartilage grafts can enhance the structural integrity of the septal L-strut. Furthermore, adjusting the cutting angle and fillet angle features can significantly influence the center of gravity and overall septal strength. The septal support graft technique demonstrated stronger support compared to other designs. The improved design concepts, involving widening the caudal septum and maximizing the size of the fillet interior corner, exhibited enhanced structural strength and stability. These design principles can be applied to septal L-struts of various dimensions, particularly when a larger septal cartilage size is not necessary. Additionally, it is recommended to prioritize preserving the width of the caudal septum over the dorsal septum during septal cartilage harvest.
